# Extrolites of *Aspergillus fumigatus* and Other Pathogenic Species in *Aspergillus* Section *Fumigati*

**DOI:** 10.3389/fmicb.2015.01485

**Published:** 2016-01-07

**Authors:** Jens C. Frisvad, Thomas O. Larsen

**Affiliations:** Section of Eukaryotic Biotechnology, Department of Systems Biology, Technical University of DenmarkKongens Lyngby, Denmark

**Keywords:** *Aspergillus*, gliotoxin, fumagillin, extrolites, proxy-exometabolites

## Abstract

*Aspergillus fumigatus* is an important opportunistic human pathogen known for its production of a large array of extrolites. Up to 63 species have been described in *Aspergillus* section *Fumigati*, some of which have also been reliably reported to be pathogenic, including *A. felis, A. fischeri, A. fumigatiaffinis, A. fumisynnematus, A. hiratsukae, A. laciniosus, A. lentulus, A. novofumigatus, A. parafelis, A. pseudofelis, A. pseudoviridinutans, A. spinosus, A. thermomutatus*, and *A. udagawae*. These species share the production of hydrophobins, melanins, and siderophores and ability to grow well at 37°C, but they only share some small molecule extrolites, that could be important factors in pathogenicity. According to the literature gliotoxin and other exometabolites can be contributing factors to pathogenicity, but these exometabolites are apparently not produced by all pathogenic species. It is our hypothesis that species unable to produce some of these metabolites can produce proxy-exometabolites that may serve the same function. We tabulate all exometabolites reported from species in *Aspergillus* section *Fumigati* and by comparing the profile of those extrolites, suggest that those producing many different kinds of exometabolites are potential opportunistic pathogens. The exometabolite data also suggest that the profile of exometabolites are highly specific and can be used for identification of these closely related species.

## Introduction

The genus *Aspergillus* comprises 344 species (Samson et al., 2014), and some of these can cause human diseases. *A. fumigatus* is the most important species (Latgé, 1999), but several other species in *Aspergillus* section *Fumigati* have been shown to be pathogenic in humans and animals with an inefficient immune system, including *A. lentulus* (Balajee et al., 2005a; Alhambra et al., 2008; Alcazar-Fuoli et al., 2014; Howard, 2014), *A. fumisynnematus* (Alcazar-Fuoli et al., 2014), *A. fumigatiaffinis* (Alcazar-Fuoli et al., 2014), *A. novofumigatus* (Peláez et al., 2013), *A. felis* (Barrs et al., 2013), *A. fischeri* (Kano et al., 2015), *A. viridinutans* (Vinh et al., 2009a; Coelho et al., 2011; Alcazar-Fuoli et al., 2014), *A. pseudofelis, A. pseudoviridinutans*, and *A. parafelis* (Sugui et al., 2014), *A. thermomutatus* (Toskova et al., 2013; Alcazar-Fuoli et al., 2014; Howard, 2014; Khare et al., 2014), *A. laciniosus* (Malejczyk et al., 2013), *A. hiratzukae* (Guarro et al., 2002; Alcazar-Fuoli et al., 2014), *A. spinosus* (Sutton et al., 2002); and *A. udagawae* (Kano et al., 2008; Vinh et al., 2009b; Sugui et al., 2010; Posteraro et al., 2011; Gyotoku et al., 2012; Kano et al., 2013). The taxonomy and identification of the causing Aspergilli is not always clear-cut and some isolates have been misidentified (Balajee et al., 2005a,b, 2006; Álvarez-Pérez et al., 2014; Howard, 2014). For example pathogenic isolates identified as *A. viridinutans* (Varga et al., 2000; Vinh et al., 2009a; Kano et al., 2013) proved to be *A. felis, A. pseudoviridinutans, A. parafelis*, or *A. pseudofelis* (Barrs et al., 2013; Novaková et al., 2014; Sugui et al., 2014). *Aspergillus* species in subgenus *Circumdati* have also been reported as pathogenic including *Aspergillus terreus* in section *Terrei, A. flavus* in section *Flavi* and *A. tubingensis* in section *Nigri, A. persii*, and *A. tanneri* in section *Circumdati, A. nidulans* in section *Nidulantes*, (Sugui et al., 2012, 2015; Howard, 2014; Visagie et al., 2014) and *Aspergillus* section *Phialosimplex* [*Ph. caninus* = *Aspergillus caninus* and *Ph. salinarum* = *Aspergillus salinarus* (Sigler et al., 2010; Greiner et al., 2014)]. Small molecule extrolites (secondary metabolites) have been shown to be involved in the infection process (Kamei and Watanabe, 2005; Abad et al., 2010), so it might be expected that the pathogenic Aspergilli produce the same extrolites. In this review we examine whether the closely related pathogenic species in *Aspergillus* section *Fumigati* produce the same extrolites.

## *Aspergillus* taxonomy

Since 2011, all ascomycetous species can only have one name (Hawksworth et al., 2011; Hawksworth, 2012; McNeill et al., 2012). All species formerly included in *Dichotomomyces, Cristaspora, Phialosimplex, Polypaecilum*, in addition to *Penicillium inflatum*, have been formally combined into *Aspergillus* (Houbraken et al., 2014; Samson et al., 2014). Furthermore, all species of *Eurotium, Emericella, Chaetosartorya, Fennellia, Neocarpenteles, Neopetromyces, Neosartorya, Petromyces, Saitoa*, and *Stilbothamnium* have also been transferred to *Aspergillus* (Samson et al., 2014). Ascoma producing species in section *Fumigati* were originally described under the name *Neosartorya* (Samson et al., 2006, 2007), but have now all been transferred to *Aspergillus* (Samson et al., 2014). Several of the species originally thought to produce only the asexual state have later been shown to be able to produce mature ascomata when crossed with the opposite mating type, for example *A. fumigatus* (O'Gorman et al., 2009) and *A. lentulus* (Swilaiman et al., 2013). Other opportunistically pathogenic species such as *A. flavus* (Horn et al., 2009), *A. tubingensis* (Horn et al., 2013), and *A. terreus* (Samson et al., 2011; Arabatsis and Velegraki, 2013) can also produce mature ascomata when crossed with the opposite mating type. All species in *Aspergillus* and *Penicillium* have now been placed in the family *Aspergillaceae* (Houbraken and Samson, 2011). Species in *Aspergillus* section *Fumigati* are both phenotypically and genotypically distinct (Raper and Fennell, 1965; Geiser et al., 1998; Hong et al., 2005, 2006, 2008; Katz et al., 2005; Geiser et al., 2007; Samson et al., 2007; Yaguchi et al., 2007). *Aspergillus lentulus* was originally claimed to be a sibling species of *A. fumigatus*, but was later shown to be phenotypically very different from *A. fumigatus*, especially concerning extrolite profiles (Larsen et al., 2007; Tamiya et al., 2015). The species *A. pseudofelis, A. parafelis*, and *A. pseudoviridinutans* have not been examined chemically, but they are very close phylogenetically and morphologically to *A. felis* and may be real sibling species with no phenotypic differences (Sugui et al., 2014). The 63 species listed in Table 1 are all those that have been described in *Aspergillus* section *Fumigati* and *Neosartorya*, but some of them are not yet available for the scientific community, so their identity and probably synonymy with other species is unknown. Samson et al. (2007) indicated that several species were synonyms of already known species in *Aspergillus* section *Fumigati* and *Neosartorya*. Thus the total number of species in *Fumigati* may be less than 63.

**Table 1:** **Species in ***Aspergillus*** section ***Fumigati*** and their extrolite production (species written in bold are known to be pathogenic to humans and/or other mammals)**.

*Aspergillus arcoverdensis*: N.E. (Matsusawa et al., 2015)
*Aspergillus assulatus*: aszonapyrone A, indole alkaloids and apolar metabolites (Samson et al., 2007)
*Aspergillus auratus*: helvolic acid (Samson et al., 2007)
*Aspergillus aureolus*: fiscalins, fumagillin, fumiquinazolines, helvolic acid, pseurotin A, tryptoquivalines, tryptoquivalones, viriditoxin (Samson et al., 2007; Kaur et al., 2013)
*Aspergillus australensis*: aszonalenins, wortmannins (Samson et al., 2007)
***Aspergillus beijingensis***: N.E. (Li et al., 1998)
*Aspergillus botucatensis* (= ***A. spinosus***) (Horie et al., 1995; Samson et al., 2007)
*Aspergillus brevipes*: roquefortine C, cf. meleagrin, viriditoxin (trace) (Lillehoj and Milburn, 1973; Samson et al., 2007)
*Aspergillus brevistipitatus*: N.E. (Novaková et al., 2014)
*Aspergillus caatingaensis*: N.E. (Matsusawa et al., 2014b)
*Aspergillus conversis*: N.E. (Novaková et al., 2014)
*Aspergillus “coreanus*” (*Neosartorya coreana*): aszonalenins (Samson et al., 2007)
*Aspergillus delicatus* (= *A. tatenoi*) (Samson et al., 2007)
*Aspergillus denticulatus*: gliotoxin, viriditoxin (Samson et al., 2007)
*Aspergillus duricaulis*: asperdurin, asperpentyn, cyclopaldic acid, duricaulic acid, fumagillin, 3-O-methylcyclopolic acid, furochromanols and phthalides, pseurotin A (Brillinger et al., 1978; Achenbach et al., 1982a,b, 1985a,b; Mühlenfeld and Achenbach, 1988a,b; Samson et al., 2007)
***Aspergillus felis***: fumagillin, fumigaclavine C, fumitremorgin A and C, helvolic acid, monomethylsulochrin, pyripyropen A, E, O, S, trypacidin (reported as “*A. viridinutans*”, Tamiya et al., 2015, but *A. viridinutans* has a very different profile of extrolites, and many isolates reported as *A. viridinutans* have been shown to be *A. felis*; Barrs et al., 2013)
*Aspergillus fennelliae*: asperfuran, aszonalenins, fumigaclavines, viridicatumtoxin (Samson et al., 2007)
*Aspergillus ferenczii*: asperfuran, aszonalenins, fumigaclavine, fumigatins, cf. gliotoxin, viridicatumtoxin (Samson et al., 2007)
***Aspergillus fischeri***: 5-N-acetylardeemin, 5-N-acetyl-15b-didehydroardeemin, 5-N-acetyl-16-hydroxyardeemin, acetylaszonalenin, ardeemin, aszonalenin, aszonapyrone A, B, cottoquinazolin E & F, cyclotryprostatin B, 12α,13α-dihydroxyfumitremorgin C, *rel*-(8S)-19,20-dihydroxy-8-methoxy-9,18-*epi*fumitremorgin C, fiscalin A, B, C, fischerin, 1-formyl-5-hydroxyaszonalenin, fumitremorgin A, B, C, helvolic acid, 6-hydroxyaszonalenin, 15b-β-hydroxy-5-N-ardeemin, isoterrein, neofipiperazine A, B, C, neosartorin, nortryptoquivalone, 13-oxofumitremorgin B, pyripyropene A, pyripyrone S, sarcins, sartorypyrone B & D, sesterfischeric acid, sesterfischerol, terrein, TR-2, trypacidin, tryptoquivalines, verruculogen (Samson et al., 1990; Wong et al., 1993; Wakana et al., 2006; Samson et al., 2007; Yin et al., 2009; Eamvijarn et al., 2013a; Lee et al., 2013; Gomes et al., 2014; Shan et al., 2014; Sodngam et al., 2014) (as “*Xylaria humosa*”) (Zhang et al., 2014; Zheng et al., 2014; Kaifuchi et al., 2015; Shan et al., 2015; Ye et al., 2015). There are indications that *A. fischeri* can also produce fumagillin (Lin et al., 2013; Wiemann et al., 2013)
[fiscalin B, helvolic acid, helvolinic acid, 27-*epi*-nortryptoquivaline, setosusin, 2-(1-oxo-2-hydroxyethyl)furan, 27-*epi*-tryptoquivaline, was found in “*Corynascus setosus*,” which is probably an *Aspergillus fischeri* or alternatively the *Corynascus* culture was overgrown by *A. fischeri*; Fujimoto et al., 1996]
[cladoquinazoline, *epi*-cladoquinazoline, CS-C, deoxynortryptoquivaline, deoxytryptoquivaline, glyantrypine, 3-hydroxyglyantrypine, norquinadoline A, oxoglyantrypine, prelapatin B, quinadoline A, B, tryptoquivaline was reported from a *Cladosporium* sp., but the culture may have been overgrown with an *Aspergillus fischeri*; Peng et al., 2013]
***Aspergillus fumigatiaffinis***: auranthine, cycloechinuline, fumigaclavines, helvolic acid, neosartorin, palitantin, pyripyropen A, E, O, S, tryptoquivalins (Samson et al., 2007; Ola et al., 2014)
***Aspergillus fumigatus***: Wang compound 1,2, 3, Zhao compound 1, 2, 3, Zuck compound 1,2,3, N-acetyltyramine, asperfumigatin, asperfumin, asperfumoid, azaspirene, bisdechlorogeodin, chaetominine, bisdethio(bismethylthio)gliotoxin, brevianamide F, 4-carboxy-5,5′-dihydroxy-3,3′-dimethyldiphenylether, cephalimycin A, B, C, D, 2-chloro-1,3,8-trihydroxy-6-methyl-9-anthrone, cyclo-(Ala-Val), cyclotryprostatin A, B, C, D, cyclo(L-4-hydroxyproline-L-leucine), cyclo(L-4-hydroxyproline-L-phenylalanine), cyclo(L-Pro-L-Pro), cyclo(L-Pro-L-Gly), cyclo(L-Pro-L-Leu), cyclo(L-Pro-L-Pro), cyclo(L-Pro-L-Val), cyclo(L-Val-L-Leu), cyclotrypostatin C, 9-deacetoxyfumigaclavine C, 9-deacetylfumigaclavine C, 13-dehydroxycyclotryprostatin C, demethoxyfumitremorgin C, (4S,5S,6S,8S,9S,10R,13R,14S,16S,17Z)-6,16-diacetoxy-25-hydroxy-3,7-dioxy-29-nordammara-1,17(20)-dien-21-oic acid, didehydrobisdethiobis(methylthio)gliotoxin, difructosedianhydride, 1,2-dihydrohelvolic acid, 12,13-dihydroxyfumitremorgin C = TR-3, 2,3-dihydroxy-5-methyl-1,4-benzoquinone, 5,8-dihydroxy-9,12-octadecadienoic acid, 2,6-dihydroxyphenylacetic acid, dimethoxyfumitremorgin C, emodin, emodin 1,6-dimethylether, epoxysuccinic acid, ferrichrome C, festuclavine, FD-889, FK-463, fumagillin, fumagiringillin, fumifungin, fumigaclavine A, B, C,D, E, F, G, H (fumigaclavine A reported also from *A. tamarii*, but this was an *A. fumigatus*, Janardhanan et al., 1984), fumigatin, fumigatin chlorohydrin, fumigatin oxide, fumigatin quinol, fumigatonin (identity of producer not verified), fumigatoside B, C, and D, fumipyrrole, fumiquinazolin A, B, C, D, E, F, G, J, and K, fumiquinone A and B, fumitremorgin A, B, C, and D, (GERI-BP002-A), fusarinine C, glionitrin A and B, gliotoxin, gliotoxin E and G, helvolic acid, helvolinic acid, hexahydropolyprenol-18, 19, 20, 21, 22, 23, 24, 3-β-hydroxy-cyclo-L-tryptophyl-L-proline, 2-hydroxy-3-methoxy-5-methyl-1,4-benzoquinone, N-(2-(4-hydroxyphenyl)ethenyl)formamide, 14-hydroxyterezine D, 3-hydroxytoluquinone, 20-hydroxytryrpostatin B, isochaetominin, isorhodoptilometrin, LL-S490β, 6-methoxyspirotryprostatin B, 8′-O-methylasterric ac id, 11-O-methylpseurotin, monomethylsulochrin, orsellinic acid, 13-oxofumitremorgin B, 18-oxotryprostatin A, 13-oxo-verruculogen, 14-norpseurotin A, N-prenyl-cyclo-L-tryptophyl-L-proline, pseurotin A, A1, A2, D, F1, F2, pyripyropen A, B, C, D, E, F, G, H, I, J, K, L, M, N, O, P, Q, R, and S, questin, RK-95113, *cis* and *trans-*ruakuric acid (Cutler et al., 1996, identity of fungus could not be checked), Sch-528647, sphingofungin A, B, C, D, spinulosin, spinulosin hydrate, spinulosin quinol hydrate, spirotryprostatin A, B, C, D, and E, synerazol, terezine D, TR-2, 1,2,3,4-tetrahydroxy-5-methylbenzene, 4,8,10,14-tetramethyl-6-acetoxy-14-[16-acetoxy-19-(20,21-dimethyl)-18-ene]phenanthrene-1-ene-3,7-dione, 3-thiomethyl-cyclo(Ser, Phe), triacetylfusarinine, tryptostatin A, B, trypacidin, 1,2-*seco*-tryprostatin, tryptoquivaline F, G, H, I, J, L, M, and N, tryptoquivalin R?, S?, verruculogen (Samson et al., 1990; Land et al., 1993; Cui et al., 1996; Tepsic et al., 1997; Furtado et al., 2005; Han et al., 2007a,b; Samson et al., 2007; Wang et al., 2008; Zhang et al., 2008 (as “*A. sydowi*”); Frisvad et al., 2009; Zhao et al., 2010; Afiyatullov et al., 2012; Zhang et al., 2012; Cano et al., 2013; Ding et al., 2013; Zhou et al., 2013; Alcazar-Fuoli et al., 2014; Haas, 2014; Kim et al., 2014; Owens et al., 2014; Wiekmann et al., 2014; Xu et al., 2014; Liang et al., 2015; Liu et al., 2015; MacHeleidt et al., 2015; Tamiya et al., 2015; Xie et al., 2015)
[*Aspergillus fumigatus* (fungus misidentified): antafumicin A and B, cytochalasin E, expansolide A and B Macías et al., 2003, the strain used was misidentified and was an *Aspergillus clavatus*; isosclerone Li et al., 2014]
***Aspergillus fumisynnematus***: cyclopiazonic acid, fumimycin, neosartorin, pyripyropens (Kwon et al., 2007; Samson et al., 2007)
*Aspergillus galapagensis*: gregatins (Samson et al., 2007)
***Aspergillus hiratsukae***: avenaciolide (Samson et al., 2007)
*Aspergillus huiyaniae*: N.E. (Matsusawa et al., 2014a)
*Aspergillus indohii*: N.E. (Horie et al., 2003)
***Aspergillus laciniosus***: aszonalenins, aszonapyrone A and B, 3′-(4-oxoquinazolin-3-yl)spiro[1H-indol-3,5′-oxolane]2,2′-dione, 4(3H)-quinazoline, tryptoquivaline L & T (Samson et al., 2007; Eamvijarn et al., 2013a; Gomes et al., 2014)
***Aspergillus lentulus***: auranthine, cyclopiazonic acid, fumifungin, fumigaclavine A, B, C, fumiquinazoline F or G, monomethylsulochrin, neosartorin, pyripyropen A, E, O, S, sphingofungin A, B, C, D, terrein, trypacidin (Larsen et al., 2007; Samson et al., 2007,: Frisvad et al., 2009; Tamiya et al., 2015)
*Aspergillus marvanovae*: apolar indoloterpenes (Hubka et al., 2013)
*Aspergillus multiplicatus*: aszonapyrone A, helvolic acid (Samson et al., 2007)
*Aspergillus neoglaber*: asperpentyn, avenaciolide, glabramycin A, B, C, Mer-NF8054A, Mer-NF8054X, NK-372135A, B, C, sartoryglabrin A, B, C, wortmannins (Ellis et al., 1964; Morino et al., 1994; Samson et al., 2007; Jayasuriya et al., 2009; Kijjoa et al., 2011)
*Aspergillus nishimurae*: Anishidiol, 4-hydroxybenzaldehyde, 4-methylbenzylalcohol, monochaetin (Hosoe et al., 2011)
***Aspergillus novofumigatus***: Dihydroterrein, epi-aszonalenin A, B, C, ent-cycloechinulin, dihydroterrein, fiscalins, helvolic acid, neosartorin, novoamauromin, novobenzomalvin A, B, C, novofumigatamide, novofumigatonin, palitantin, terrein, territrem B (Rank et al., 2006; Samson et al., 2007; Rank et al., 2008; Hosoe et al., 2009; Ishikawa et al., 2010a,b, 2011)
*Aspergillus otanii* = *A. fennelliae* (Takeda et al., 2001; Samson et al., 2007)
*Aspergillus papuensis*: wortmannins (Samson et al., 2007)
***Aspergillus parafelis***: N.E. (Sugui et al., 2014)
*Aspergillus paulistensis* [= *A. spinosus* according to Samson et al. (2007)]: 3′-(4-oxoquinazolin-3-yl)spiro[1H-indol-3,5′-oxolane]2,2′-dione, 4(3H)-quinazoline, sartorypyrone C, tryptoquivaline L (Horie et al., 1995; Gomes et al., 2014)
*Aspergillus pernambucoensis*: N.E. (Matsusawa et al., 2014b)
*Aspergillus primulinus = A. quadricinctus* (Samson et al., 2007)
***Aspergillus pseudofelis***: N.E. (Sugui et al., 2014)
*Aspergillus pseudoviridinutans*: N.E. (Sugui et al., 2014)
*Aspergillus quadricinctus*: aszonalenins, PF1223, quinolactacin (Ozoe et al., 2004; Samson et al., 2007)
***Aspergillus qizutongii***: N.E. (Li et al., 1998)
*Aspergillus shendawei*: N.E. (Yaguchi et al., 2010)
*Aspergillus siamensis*: chevalone B, C, 4-dihydroxy-3-methylacetophenone, fiscalin A, C, *epi*-fiscalin A, C, neofiscalin A, *epi-*neofiscalin A, 3′-(4-oxoquinazolin-3-yl)spiro[1H-indole-3,5′-oxolane]-2,2′-dione, sartorymensin, tryptoquivaline, tryptoquivaline F, H, L, O (Buttachon et al., 2012; Eamvijarn et al., 2013b)
*Aspergillus similanensis*: chevalone, B, C, E, 6,8-dihydroxy-3,7-dimethylisocoumarin, 6,8-dihydroxy3-methylisocoumarin, p-hydroxybenzaldehyde, 5-hydroxy-8-methyl-2H,6H-pyrano[3,4-g]chromene-2,6-dione, pyripyropen E, S, and T, reticulol, S14-95, similanamide, similanpyrone C (Prompanya et al., 2014, 2015)
*Aspergillus solicola*: aszonalenins, chromanols, tryptoquivalines, tryptoquivalones, wortmannins (Samson et al., 2007, 2014)
*Aspergillus spathulatus*: aszonalenins, xanthocillins (Samson et al., 2007)
***Aspergillus spinosus***: aszonalenins, pseurotins, 2-pyrovoylaminobenzamide, fumigachlorin (Atsumi et al., 1970; Samson et al., 2007)
*Aspergillus stramenius*: avenaciolide, quinolactacin (Samson et al., 2007)
*Aspergillus sublevisporus*: N.E. (Someya et al., 1999)
*Aspergillus takakii* = *A. spinosus* (?) (Samson et al., 2007): acetylaszonalenin, aszonalenin, aszonapyrone A, chevalone B, 6-hydroxymellein, 3′-(4-oxoquinazolin-3-yl) spiro[1H-indole-3,5′-oxalone]-2-2′-dione, tryptoquivaline F, H, L, U (Zin et al., 2015)
*Aspergillus tatenoi*: aszonalenin, aszonapyrone A, B, tatenoic acid (Samson et al., 2007; Yim et al., 2014)
***Aspergillus thermomutatus***: 6-acetylbis(methylthio)gliotoxin, acetylgliotoxin, asperfuran, bisdethiobis(methylthio)gliotoxin, bis-N-norgliovictin, brasilianamide B, cadinene, CJ-12662?, 3,8-diacetyl-4-(3-methoxy-4,5-methylenedioxy)benzyl-7-phenyl-6-oxa-3,8-diazabicyclo[3.2.1]octane, didehydrobisdethiobis(methylthio)gliotoxin, euchevalierine, fiscalins (?), fischerindoline, gliotoxin, helvolic acid, 3-hydroxy-5-methylphenyl-2,4-dihydroxy-6-methylbenzoate, N-methyl-1H-indole-2-carboxamide, neosartorin A, B, C, pseudofischerine, pyripyropen A, E, O, S, 1,2,3,4-tetrahydro-2,3-dimethyl-1,4-dioxopyrazino[1,2-a]indole, 1,2,3,4-tetrahydro-2-methyl-3-methylene-1,4-dioxopyrazino[1,2-a]indole, 1,2,3,4-tetrahydro-2-methyl-1,3,4-trioxopyrazino[1,2-a]indole, (tryptoquivalin R, S ?) [maybe: misidentified as “*Eurotium chevalieri*”: cadinene, chevalone A, B, C, D, aszonapyrone A, B, euchevalerine, CJ-12662 Kanokmedhakul et al., 2011] (Samson et al., 2007; Eamvijarn et al., 2012; Masi et al., 2013; Xu et al., 2013; Liang et al., 2014)
*Aspergillus tsunodae*: helvolic acid, sartorypyrone A and B (Yaguchi et al., 2010; Eamvijarn et al., 2013a; Gomes et al., 2014)
*Aspergillus tsurutae*: N.E. (Horie et al., 2003)
*Aspergillus turcosus*: aszonalenins, gliotoxin, kotanins (Samson et al., 2007; Hubka et al., 2013)
***Aspergillus udagawae***: fumagillin, fumigaclavine A and C, fumigatins, fumiquinazolin F or G, helvolic acid, monomethylsulochrin, pyripyropene A, E, trypacidin, tryptoquivalines, tryptoquivalones (Samson et al., 2007; Tamiya et al., 2015)
*Aspergillus unilateralis*: aszonapyrones, mycophenolic acid (Samson et al., 2007; Hubka et al., 2013)
*Aspergillus viridinutans*: 4-acetyl-6,8-dihydroxy-5-methyl-2-benzopyran-1-1 A, 13-O-methylviriditin, phomaligin A, SC-28763, SC-30532, semiviriditoxin, viriditoxin, viritin, viriditin, wasabidienone B0 and B1 (Omolo et al., 2000; Samson et al., 2007)
*Aspergillus waksmanii*: apolar indoloterpenes (Hubka et al., 2013)
***Aspergillus wangduanglii***: N.E. (Li et al., 1998)
*Aspergillus wyomingensis*: N.E. (Novaková et al., 2014)

*N.E: Not Examined*.

## Chemotaxonomy of *Aspergillus* subgenus *Fumigati*

Species in subgenus *Fumigati* can produce many different extrolites (Frisvad and Samson, 1990; Samson et al., 2007; Stack et al., 2007; Varga et al., 2007; Frisvad et al., 2009; Sanchez et al., 2012; Kang et al., 2013; Frisvad and Larsen, 2015) of which some are specific to section *Fumigati*, while others are shared with the closely related section *Clavati* and the *Dichotomomyces* clade. *Aspergillus cejpii* in the *Dichotomomyces* clade produces gliotoxin, acetylgliotoxin, acetylgliotoxin G, bis(dethio)bis(methylthio)gliotoxin, fiscalin B, xanthocillin X monomethylether, tryptoquivalones, emindole SB, emindole SB β-mannoside, and 27-O-methylasporyzin (Varga et al., 2007; Harms et al., 2014; Rodrigues et al., 2015) possibly in addition to asporyzin A-C, emeniveol, JBIR-03, and asporyergosterol and other sterols (Qiao et al., 2010a,b). The producing strain of the latter exometabolites was probably misidentified as *A. oryzae*, since none of these exometabolites have ever been found in *A. oryzae* (Rank et al., 2012). Apart from some few other shared extrolites with *Aspergillus* species in other sections, most extrolites are unique to section *Fumigati*.

*Aspergillus* section *Clavati* contains species mostly associated to dung, and have not been reported to cause infections of vertebrate lungs (Varga et al., 2007). Species in *Aspergillus* section *Clavati* produce several bioactive extrolites, but few of these are found in *Aspergillus* section *Fumigati*. Examples of such *Aspergillus* section *Clavati* specific extrolites include patulin, cytochalasin E and K, antafumicins, expansolides, and clavatols, and these extrolites may be important for competition in a dung habitat, rather than in the compost habitats in which species of *Aspergillus* section *Fumigati* thrives. Some similar extrolites are in common between species in *Aspergillus* sections *Fumigati* and *Clavati*, however. Ribotoxins like the sarcins in *Aspergillus* section *Clavati* (Varga and Samson, 2008) are closely related to mitogillin and restrictocin in *Aspergillus* section *Fumigati* (Kao et al., 2001; Schwienbacher et al., 2005; Virágh et al., 2014). Furthermore, some tryptoquivalins are produced by species in both *Aspergillus* sections.

Like other filamentous fungi, *A. fumigatus* isolates produce extrolites in a species specific manner (Larsen et al., 2005; Frisvad et al., 2008), but some strains do not produce all the extrolites expected. This weaker exometabolic vigor is most pronounced in isolates directly isolated from patients (Frisvad and Samson, 1990; Tamiya et al., 2015). These isolates are often floccose and less strongly sporulating. However, isolates from natural habitats, such as compost, always sporulate heavily and produce most of the expected species specific extrolites (Frisvad and Samson, 1990; Tepsic et al., 1997; Hong et al., 2010a,b). Production of small molecule extrolites is depending on the growth conditions and the growth media (Nielsen et al., 2011; Frisvad, 2012; Brakhage, 2013), and some of these extrolites may need biological / chemical stimulants of the producing fungus to be expressed (Brakhage and Schroeckh, 2011; Zuck et al., 2011; Netzker et al., 2015).

Being species specific, the difference between the extrolites profiles of different species of *Aspergillus* section *Fumigati* can be used in identification of the species in *Aspergillus* section *Fumigati* as an alternative to sequence—based or MALDI-TOF based identification (Panda et al., 2015), or used together with morphology and physiology in a polyphasic identification approach (Samson et al., 2007). For example *A. fumigatus* can be distinguished from *A. lentulus* by exometabolite profiling (Larsen et al., 2007), MALDI-TOF (Verwer et al., 2014), and sequencing (Balajee et al., 2005a; Samson et al., 2007), but only partially by morphology and Raman spectroscopy (Verwer et al., 2014).

## Extrolites produced by *Aspergillus fumigatus* and other pathogenic species in *Fumigati*

*A. fumigatus* has been reported to produce many different extrolites that are bioactive and may contribute to infection in humans and other animals (Amitani et al., 1995; Tomee and Kauffman, 2000; Reeves et al., 2006; Cramer et al., 2009; Abad et al., 2010; Coleman et al., 2011). Melanins are polyketide derived conidium pigments that may have an influence on the infection process (Tsai et al., 1998; Jahn et al., 2000; Tsai et al., 2001; Langfelder et al., 2003). Since all species in *Aspergillus* section *Fumigati* produce green conidia, it is expected that they all contain melanin (Perrin et al., 2007). Another more general small molecule pathogenicity factor is siderophores, of which *A. fumigatus* produces fusarinine C and triacetylfusarinine C extracellularly (Haas, 2014; Petrik et al., 2014). Furthermore hydrophobins are also present in all species of *Aspergillus* section *Fumigati* (Geiser et al., 1998; Pedersen et al., 2011). These proteins will protect conidia from being recognized by the immune system in mammals (Aimanianda et al., 2009). Other proteins, especially proteases also play a role in the infection process and may be expected to be produced by many pathogenic species (Tomee and Kauffman, 2000; Abad et al., 2010; Dhingra et al., 2012). Small molecule siderophores are also considered to be important pathogenicity factors, and given the general importance for fungi they can be expected to be produced by all pathogenic species of *Aspergillus* (Fedorova et al., 2008; Abad et al., 2010; Haas, 2014), but probably also by non-pathogenic species.

However, other extrolites are not produced by all species in *Aspergillus* section *Fumigati*. Gliotoxin has long been known to be important for the infection process by inhibiting the immune response, phagocytosis and angiogenesis (Watanabe et al., 2003, 2004; Tsunawaki et al., 2004; Bok et al., 2005; Lewis et al., 2005; Stanzani et al., 2005; Coméra et al., 2007; Sugui et al., 2007; Ben-Ami et al., 2009; Abad et al., 2010). Gliotoxin has been reported from the pathogenic species *A. fumigatus* and *A. thermomutatus*, but also from *A. denticulatus, A. ferenczii* and *A. turcosus* (Table 1) the latter three not yet known to be pathogenic. Annotation of the genomes of *A. fumigatus* and *A. fischeri* indicates that the latter species can also produce gliotoxin given the right conditions (Inglis et al., 2013). However, many other *Aspergillus* section *Fumigati* extrolites appear to be involved in pathogenesis. Verruculogen, produced by *A. fumigatus* and *A. fischeri*, modifies electrophysical properties of the human nasal epithelial cells (Khoufache et al., 2007) but is also a potent tremorgen (Land et al., 1993; Kelman et al., 2004). Verruculogen and fumitremorgin C (Rabindran et al., 2000) are produced by *A. fumigatus* and *A. fischeri* (Table 1) in section *Fumigati*. Fumagillin suppresses the immune response, neutrophil function and angiogenesis (Fallon et al., 2010, 2011) and is produced by the pathogenic species *A. felis, A. fumigatus*, and *A. udagawae*, but also by species in *Aspergillus* section *Fumigati*, such as *A. aureolus* and *A. viridinutans* that have not been reported as yet to be pathogenic (Table 1). Pseurotin A is an inhibitor of immunoglobulin E and is responding to hypoxia (Schmeda-Hirschmann et al., 2008; Ishikawa et al., 2009; Vödisch et al., 2011). Pseurotins are produced by the pathogenic *A. fumigatus* and *A. spinosus*, but are also produced by *A. duricalis* and *A. aureolus* (Table 1). Sulochrin inhibits eosinophil activation (Ohashi et al., 1997, 1998) and is produced by four pathogenic species in section *Fumigati*: *A. felis, A. fumigatus, A. lentulus*, and *A. udagawae* (Table 1). The related asterric acid is produced by the same species and this extrolite inhibits vascular endothelial growth factor induced tube formation (Lee et al., 2013). Another related extrolite is trypacidin, which is cytotoxic (Gauthier et al., 2012), but was originally isolated as an antiprotozoan metabolite (Balan et al., 1963). The fumiquinazolins are also cytotoxic (Lim et al., 2014), and are produced consistently by *A. fumigatus* (Frisvad et al., 2009). The fumiquinazolines (Takahashi et al., 1995) are produced by the pathogenic *A. fumigatus* and *A. lentulus*, but are also produced by *A. aureolus* (Table 1). The chemically similar fiscalins and cottoquinazolins (norfumiquinazolins; Ames and Walsh, 2010; Shan et al., 2015) are produced by *A. fischeri*, indicating that these metabolites are of importance for the competitiveness of these fungi. The pyripyropenes have antiangiogenic activity (Hayashi et al., 2009) and are produced by nearly all the known pathogenic species in section *Fumigati*: *A. fumigatus, A. fumigatiaffinis, A. fumisynnematus, A. lentulus, A. thermomutatus*, and *A. udagawae* (Table 1). In addition pyripyropens are produced by *A. similanensis*, a species that has not yet been tested for pathogenicity or isolated from any animal tissues.

Helvolic acid has been reported as an antibiotic and antifungal extrolite (Rementeria et al., 2005), but it also has been reported to affect human respiratory epithelium (Amitani et al., 1995) and the metabolism of macrophages (Shinohara et al., 1992). Helvolic acid has been reported from *Aspergillus auratus, A. aureolus, A. felis, A. fischeri, A. fumigatiaffinis, A. fumigatus, A. multiplicatus, A. novofumigatus, A. thermomutatus, A. tsunodae*, and *A. udagawae*. It is upregulated with gliotoxin in *A. fumigatus* (O'Keeffe et al., 2014). Thus helvolic acid may also be a pathogenicity factor, but of the species listed above *A. auratus, A. aureolus, A. multiplicatus*, and *A. tsunodae* have not been reported as pathogenic. Among bioactive proteins it seems that mitogillin is playing a role in the infection process (Schwienbacher et al., 2005; Abad et al., 2010), but these ribotoxins have not been screened in the other 62 species in *Aspergillus* section *Fumigati*.

Several small molecule extrolites have not yet been claimed to be involved in pathogenesis. The fumigaclavines are produced by the pathogenic species *A. felis, A. fumigatus, A. fumigatiaffinis*, and *A. lentulus*, but are also produced by *A. fennelliae* and *A. ferenczii* (Table 1). Even though these ergot alkaloids are associated with conidiation in *A. fumigatus* (Coyle et al., 1981), their role in animal pathogenesis is unknown. The fumigatins have mostly been found in soil-borne strains of *A. fumigatus* (Frisvad and Samson, 1990), and may rather have a role in competitiveness in compost and soil, than in animal pathogenesis.

## Prediction of other potential opportunistic pathogenic species in *Aspergillus* section *Fumigati* based on extrolites

Among the 63 species described in *Aspergillus* section *Fumigati*, 17 have until now been reported to be opportunistic pathogens of vertebrate animals (in bold, Table 1). Several extrolites have been shown to have a certain role in the infection process, but these extrolites may have a different role in the natural habitats of these fungi, of which plant compost may be the primary habitat (Latgé, 1999; Abad et al., 2010). It appears that when growing on plant compost these fungi need a certain profile of extrolites (small molecule extrolites and exoproteins), while as vertebrate opportunistic pathogens they may need quite a different profile of extrolites (Abad et al., 2010). For example cellulases would be important in the compost situation (Srivastava et al., 2014; Miao et al., 2015), while hemolysins are probably only important for the vertebrate infection process (Abad et al., 2010). The same would be the case for antifungals and antibiotics, especially anti-streptomycete metabolites, as *A. fumigatus* and other members of *Aspergillus* section *Fumigati* are thermotolerant / thermophilic species competing with other thermotolerant and thermophilic species of fungi and bacteria (Langarica-Fuentes et al., 2014). Several species, such as *A. assulatus, A. australensis, A. brevipes, A*. “*coreanus*,” *A. duricaulis, A. fennelliae, A. galapagensis, A. neoglaber, A. marvanovae, A. nishimurae, A. papuensis, A. quadricinctus, A. solicola, A. spathulatus, A. tatenoi, A. unilateralis, A. viridinutans*, and *A. waksmanii* produce few if any of the extrolites suspected to play a role in the infection process, and so would not be predicted to be potential opportunistic pathogens of vertebrates. Some species, such as *A. auratus, A. denticulatus, A. similanensis, A. tsunodae*, and *A. turcosus* only produce one of the extrolites believed to play a role in pathogenesis, and may or may not be prospective vertebrate pathogens. Finally *A. aureolus, A. ferenczii*, and *A. siamensis* produce several of the extrolites potentially involved in pathogenesis, and thus may be predicted to be potential opportunistic vertebrate pathogens.

**Figure 1:**
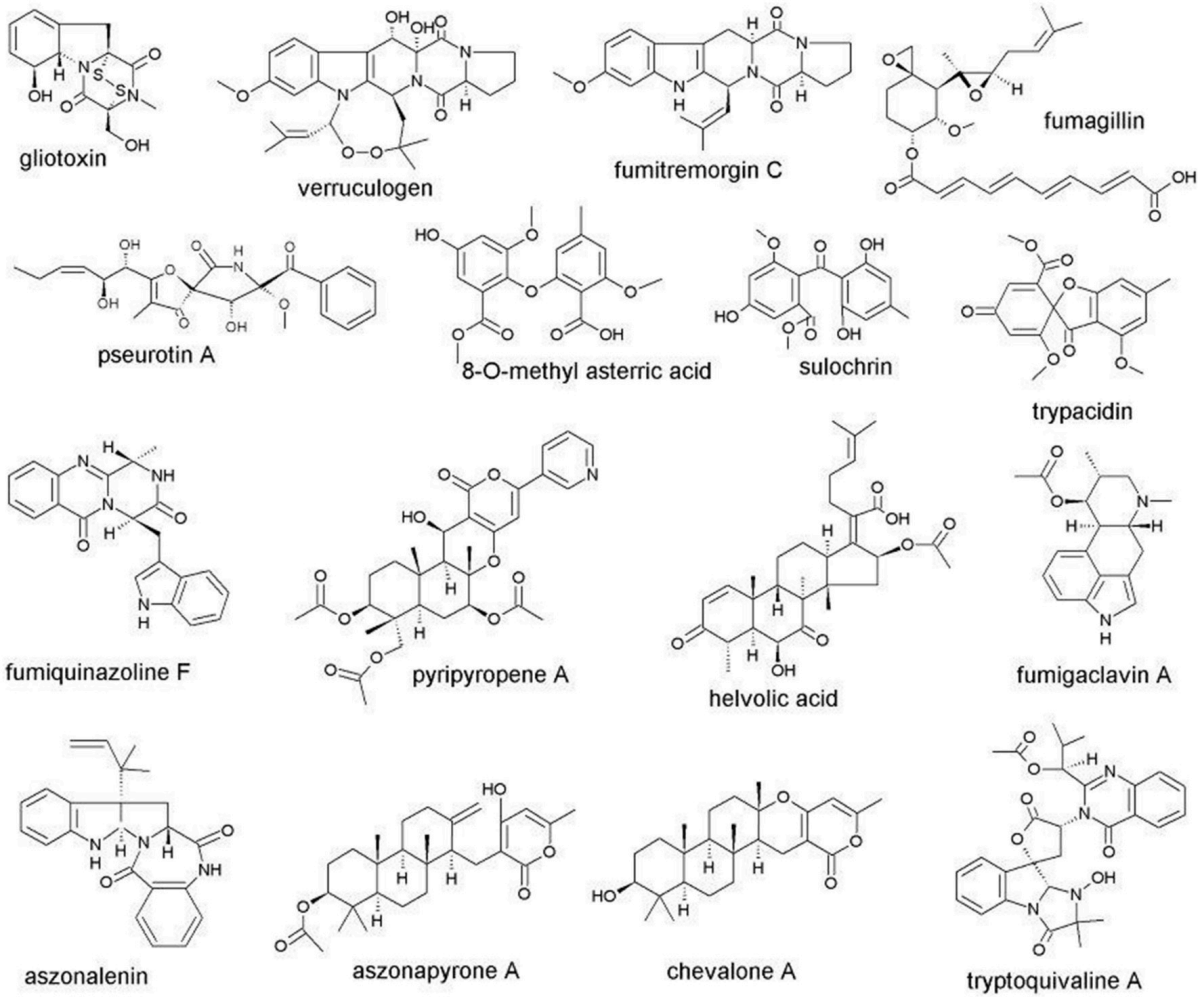
**Structures of the most important extrolites from ***Aspergillus*** section ***Fumigati*** potentially involved in pathogenesis**.

The many extrolites that have been suspected to be pathogenicity factors and are produced by species in *Aspergillus* section *Fumigati* are biosynthetically derived from polyketides, amino acids, terpenes, shikimic acid or are of mixed biosynthetic origin. The formula of some of the most important extrolites common in *Aspergillus* section *Fumigati* are shown in Figure 1. Some of the extrolites are not produced in the same patterns in different species in *Aspergillus* section *Fumigati*. While *A. fumigatus* produces fumiquinazolins A–G, J, and K, *A. fischeri* produces the related norfumiquinazolins (Shan et al., 2015). These extrolites may have the same function, even though they are chemically somewhat different. Whether such proxy-extrolites have the same function for pathogenicity in vertebrates is unknown. It is known, however, that other opportunistic pathogenic aspergilli in other sections of *Aspergillus* produce secondary metabolites that are biosynthetically and functionally closely related. While *A. fumigatus, A. thermomutatus*, and other species in section *Fumigati* produce gliotoxin, *A. flavus* in *Aspergillus* section *Flavi* can produce aspirochlorine and *A. terreus* in *Aspergillus* section *Terrei* can produce acetylaranotin (Frisvad and Larsen, 2015). While not identical to gliotoxin, these epidithiodioxopiperazines could be predicted to play a role in pathogenicity of *A. flavus* and *A. terreus*. The reports that *A. niger, A. flavus*, and *A. terreus* could produce gliotoxin (Lewis et al., 2005; Kupfahl et al., 2008) have not been confirmed (Samson et al., 2011; Varga et al., 2011a,b).

Close phylogenetic relationships seem to be less suited pathogenicity predictors. For example *A. viridinutans* seems to be non-pathogenic, while the closely related *A. felis* is pathogenic (Barrs et al., 2013; Novaková et al., 2014; Sugui et al., 2014). Good growth at 37°C also seems to be a contributing factor to pathogenicity, and for example *A. brevipes, A. duricaulis* and *A. viridinutans* grow relatively poorly at 37°C, and in addition are not considered potentially pathogenic *Aspergillus* species in section *Fumigati*, based on extrolite evidence and absence of reports of pathogenicity. However, while there a many data on the involvement of exometabolites for *A. fumigatus* (Abad et al., 2010), there are few data on production of exoproteins for other opportunistic pathogenic species such as *A. thermomutatus*.

Genome sequencing and systematic comparison of the genomes and transcriptomes of other members of *Aspergillus* section *Fumigati* may help in predicting which pathogenicity factors are especially important (Galaghan et al., 2005; Nierman et al., 2005; Wortman et al., 2006; Fedorova et al., 2008; McDonagh et al., 2008; Chooi et al., 2013; Inglis et al., 2013; Cerqueira et al., 2014; Kusuya et al., 2015; Lind et al., 2015). These data should be compared to phenotypic data such as profiles of large and small molecule extrolites, growth temperatures, carbon dioxide tolerance etc.

Altogether, approximately one third of the species in *Aspergillus* section *Fumigati* are common pathogenic species, one third are rare species of unknown pathogenicity and one third are predicted to be non-pathogenic, based on their production of relatively few exometabolites. Exometabolite pathogenicity factors found in the successful opportunistic pathogenic fungus *A. fumigatus* may have proxy-exometabolites with the same function in other species in that section, but also in less closely related pathogenic Aspergilli, especially species from sections *Nigri, Terrei, Circumdati*, and *Flavi*.

### Conflict of interest statement

The authors declare that the research was conducted in the absence of any commercial or financial relationships that could be construed as a potential conflict of interest.
